# Dangguijihwang-tang and Dangguijakyak-san Prevent Menopausal Symptoms and Dangguijihwang-tang Prevents Articular Cartilage Deterioration in Ovariectomized Obese Rats with Monoiodoacetate-Induced Osteoarthritis

**DOI:** 10.1155/2017/5658681

**Published:** 2017-10-24

**Authors:** Hye Won Lee, Byung-Seob Ko, Suna Kang, Jin Ah Ryuk, Min Joo Kim, Sunmin Park

**Affiliations:** ^1^Korea Institute of Oriental Medicine, Daejeon, Republic of Korea; ^2^Department of Food and Nutrition, Obesity/Diabetes Research Center, Hoseo University, Asan, Republic of Korea

## Abstract

We investigated whether dangguijakyak-san (DJY) and dangguijihwang-tang (DJH), oriental medicines traditionally used for inflammatory diseases, could prevent and/or delay the progression of postmenopausal symptoms and osteoarthritis in osteoarthritis-induced estrogen-deficient rats. Treated ovariectomized (OVX) rats consumed either 1% DJY or 1% DJH in the diets. Positive-control rats were given 30 *μ*g/kg bw 17*β*-estradiol and control rats were given 1% fat as were the normal-control rats. All rats received high-fat diets for 8 weeks. At the 9th week, OVX rats received articular injections of monoiodoacetate (MIA) or saline (normal control) into the right knee. At 3 weeks after MIA injection, DJY reduced visceral-fat mass and improved glucose metabolism by reducing insulin resistance, whereas DJH increased BMD and decreased insulin resistance. DJH improved weight distribution in the right knee and maximum running velocity on a treadmill at days 14 and 21 as much as those of the positive control. TNF-*α*, IL-1*β*, and IL-6 levels in articular cartilage were much higher in the control than the positive control, whereas both DJY and DJH reduced the levels to those of the positive control. The histological analysis assessed articular cartilage damage near the tidemark and proteoglycan loss in the control versus the positive control; DJY and DJH prevented this damage and proteoglycan loss. In conclusion, DJY may provide an effective treatment for improving glucose tolerance, and DJH may be appropriate for preventing osteoarthritis.

## 1. Introduction

Estrogen binds to the estrogen receptor on the nucleus to directly activate estrogen response element-mediated transcription and transcription factor cross talk [1]. Additionally, estrogen binds to an estrogen receptor in the cell membrane or cytosol to activate phosphoinositide 3-kinase (PI3-kinase) and mitogen-activated protein kinase (MAPK) signal transduction pathways to stimulate growth factors and activate the Wnt signaling pathway [1]. This estrogen signal transduction is involved in various metabolic processes, including energy, glucose, lipid, and bone metabolism [1, 2]. Estrogen deficiency impairs these various signaling pathways, resulting in the development of various metabolic disturbances. Postmenopausal women are at increased risk of metabolic conditions, such as obesity, heart disease, diabetes, osteoporosis, and hypertension [3].

In addition to metabolic disorders, estrogen deficiency elevates low-grade systematic inflammation, with increased levels of proinflammatory cytokines, such as interleukin-1 (IL-1) and tumor necrosis factor-*α* (TNF-*α*) as well as prostaglandins [4, 5]. Expression of these proinflammatory cytokines has been shown to be attenuated by estrogen replacement. A deficiency in estrogen receptors in female mice results in the development of osteoarthritis, with cartilage damage, osteophytosis, and damage in the subchondral bone of the joints [6, 7]. Estrogen deficiency increases bone resorption and it may be associated with elevated levels of proinflammatory cytokines [8]. Osteoporosis worsens osteoarthritis in menopausal women. Thus, menopause makes women more susceptible to osteoarthritis.

Hormone replacement therapy (HRT, primarily estrogen) and selective estrogen receptor modulators (SERMs) improve metabolic disorders, including obesity, type 2 diabetes, dyslipidemia, and osteoarthritis, by activating estrogen receptor-related pathways [9, 10]. However, HRT and SERMs have adverse effects. Herbs containing phytoestrogens may prevent and/or alleviate menopausal and osteoarthritis symptoms related to estrogen deficiency [9]. Herbal treatments with no or minor adverse effects need to be explored to alleviate these symptoms, possibly by stimulating PI3-kinase and MAPK signal transduction pathways [11]. Inflammation is also enhanced not only through estrogen receptors but also by direct actions on NF-*κ*B signaling [12]. Herbal treatments may alleviate osteoarthritis more than HRT and SERMs.

As* Angelicae Gigantis* Radix (Danggui,* Angelica sinensis*) is recognized as a “female ginseng,” it is used widely to reduce primary dysmenorrhea and menopausal symptoms [13]. It contains decursin, decursinol, decursinol angelate, nodakenin, n-butylidenephthalide, and umbelliferone [14]. The components have been reported to improve energy and glucose and lipid metabolism and to reduce inflammation and oxidative stress [15, 16]. Supporting the potential benefits for preventing osteoarthritis, Kil et al. [17] demonstrated that* Angelicae Gigantis* Radix inhibits the formation of osteoclasts in mouse bone marrow cells challenged with TNF-*α*. Furthermore, another study demonstrated that decursin from* Angelicae Gigantis* Radix suppresses RANKL-induced osteoclast formation [18]. Therefore, these studies provide good evidence that* Angelicae Gigantis* Radix, a major component of both DJY and DJH, may help slow bone resorption and would be expected to protect against osteoarthritis and osteoporosis.* Angelicae Gigantis* Radix may potentiate the activity with the combination of other herbs. The recognized prescriptions including* Angelicae Gigantis* Radix are Dangguijakyak-san (“Dang-Gui-Shao-Yao-San” in Chinese, “Toki-shakuyaku-san” in Japanese; DJY) and Dangguijihwang-tang (Dangguiliuhuang-tang in Chinese; DJH). Both prescriptions have been used traditionally for treating menopause-related symptoms and inflammation-associated diseases in East Asia [19–21]. DJY consists of six herbs, including* Angelicae Gigantis* Radix and* Paeoniae* Radix that have been reported to have anti-inflammatory activities [20].* Paeoniae* Radix contains albiflorin, paeoniflorin, and paeonol and they have anti-inflammatory activities [22]. DJH is composed of* Angelicae Gigantis* Radix and* Rehmanniae* Radix Preparata (1 : 2, w/w). Sukjihwang (shudihuang in Chinese), steamed and dried* Rehmanniae* Radix, has been used traditionally to make menstruation regular, reduce fatigue and anxiety symptoms, and inhibit inflammatory responses [23–25]. Sukjihwang is composed of dihydrocatalpol, danmelittoside, acetylcatalpol, leonuride, aucubin, melittoside, and rehmaglutin [26]. DJY has been shown to potently suppress prostaglandin F2-induced rat uterus muscle contractions* in vitro*, suggesting possible efficacy for treating menstrual cramps [27]. In addition, 50% ethanol extract of* Rehmanniae* Radix improves microcirculation and reduces inflammation in adjuvant-induced arthritis animal [28]. DJY and DJH may alleviate the symptoms related to menopause and osteoarthritis, although the mechanism remains unclear. However, if it is involved in the inhibition of cyclooxygenase enzymes, improvement of microcirculation, and reduction of inflammation, it could benefit inflammation-related postmenopausal symptoms as well.

The hypothesis of this study was that aqueous extracts of DJY and DJH would prevent and/or delay the progression of postmenopausal symptoms and osteoarthritis in estrogen-deficient animals. We examined this hypothesis and explored the protective mechanism against menopausal symptoms and knee joint osteoarthritis in osteoarthritis-induced ovariectomized (OVX) rats fed with a high-fat diet. Since OVX rats with MIA-induced osteoarthritis have similar phenotypes to menopausal women with moderate osteoarthritis symptoms, the rats are appropriate to use as the animal model in the present study.

## 2. Materials and Methods

### 2.1. Preparation of Aqueous Extracts of DJY and DJH

*Paeoniae* Radix,* Cnidii* Rhizoma,* Alismatis* Rhizoma,* Angelicae Gigantis* Radix,* Poria Sclerotium*,* Atractylodis* rhizome, and* Rehmanniae* Radix Preparata were purchased from Backjaedang (Daejeon, Korea). Voucher specimens (KIOM M 130002-3, KIOM M 13000 5–7, KIOM M 130010, and KIOM M 130012) were deposited at KM Convergence Research Division, Korea Institute of Oriental Medicine.

The DJY decoction was made from water extracts of* Paeoniae* Radix,* Cnidii* Rhizoma,* Alismatis* Rhizoma,* Angelicae Gigantis* Radix,* Poria Sclerotium*, and* Atractylodis* rhizome (3 : 2 : 2 : 1 : 1 : 1, w/w). The DJH decoction was produced from water extracts of* Angelicae Gigantis Radix* and* Rehmanniae* Radix Preparata (1 : 2, w/w). The herbs in the DJY and DJH were added to distilled water and boiled for 4 h at 100°C. Each extract was filtered using filter paper (Whatman, Maidstone, UK), evaporated, and lyophilized. The yields of DJY and DJH water extracts were 29.5 and 64.5%, respectively.

### 2.2. Major Components of Aqueous Extracts of DJY and DJH

A reference standard stock solution of four compounds (albiflorin, paeoniflorin, nodakenin, and 5-hydroxymethyl-2-furaldehyde) was dissolved in methanol at 1.0 mg/mL. For HPLC analysis, 46.8 and 20 mg of the lyophilized DJY and DJH water extracts, respectively, were dissolved in 1 mL distilled water, and the solutions were filtered through a 0.45 *μ*m syringe filter and then injected into the HPLC system (an Agilent 1100 series HPLC instrument, Agilent Technologies, USA, comprising an autosampler, a column oven, a binary pump, a DAD detector, and a degasser). ChemStation software (Agilent Technologies, USA) was used for processing the data. The HPLC analytical column was an Atlantis C_18_ column (250 × 4.6 mm, particle size 5 *μ*m, Waters, USA); it was maintained at 30°C. The injection volume was 10 *μ*L and the flow rate was 1.0 mL/min. For DJY, the mobile phase consisted of water in 0.1% trifluoroacetic acid (TFA, v/v) (A) and acetonitrile (B). The following gradient was used: 0–20 min, 10–25% B; 20–25 min, 25% B; 25–30 min, 25–50% B; 30–45 min, 50–80% B; 45–60 3–4 min, 80–100% B. For DJH, the mobile phase was water in 0.1% TFA (A) and acetonitrile (B) and the column was eluted with a gradient flow with 0–10 min, 5% B; 10–30 min, 5–75% B; and 30–50 min, 75–90% B. The quantitative analysis of the three marker compounds for DJY was performed at 230 nm for albiflorin and paeoniflorin and at 330 nm for nodakenin. The three markers for DJH were analyzed at 280 nm for 5-hydroxymethyl-2-furaldehyde and at 330 nm for nodakenin and decursin.

### 2.3. Ovariectomy Operation and Monoiodoacetate- (MIA-) Induced Osteoarthritis Animal Model

This research followed the guidelines of the NIH Guide for the Care and Use of Laboratory Animals and the International Association for the Study of Pain, and every effort was made to reduce pain and suffering of the animals. The research design and all procedures were approved by the Animal Care and Use Committee of Hoseo University, Korea (2014-02).

After purchasing 50 Sprague Dawley female rats aged 8 weeks (230 ± 30 g) from DBL (Yeumsung-Kun, Korea), they were acclimated for 1 week in our animal facility. The rats were housed individually in stainless steel cages in a controlled environment (23°C and with a 12/12 h light/dark cycle). After 12 h fasting, rats underwent bilateral OVX under anesthesia, induced by subcutaneous injection of a mixture of ketamine and xylazine (100 and 10 mg/kg body weight, resp.), as described previously [29, 30].

Osteoarthritis was induced by intra-articular injection of MIA. The MIA injection is known to produce a similar pathology to OA by disrupting glycolysis at the site of the injection resulting in eventual death of chondrocytes, and by increased inflammation [31]. After anesthetization with intramuscular injections of a ketamine and xylazine mixture (100 and 10 mg, resp.), 40 OVX rats received a single intra-articular injection of MIA (4 mg/50 *μ*L saline; Sigma Co.) through the patellar ligament of the right knee, using a 26-gauge needle. The remaining 10 OVX rats received a single intra-articular injection of saline into the right knee as the normal control. On the day following the MIA injections, the rats were observed for changes in behaviors and in right knee morphology by a single, trained technician. MIA injection is considered to induce osteoarthritis in joints by damaging glycolysis in the joint, resulting in the eventual death of chondrocytes by inhibiting glyceraldehyde-3-phosphate dehydrogenase activity and increasing inflammation [29, 32].

### 2.4. Diet Preparation

The dosages of dangguijakyak-san and dangguijihwang-tang were assigned on the basis of traditional human usage and conversion factor from human to animal. The dosage of each herbal prescription was about 50 mg/kg bw for human. The extract powders were mixed with a high-fat diet since the diet exacerbated the progression of menopausal symptoms and osteoarthritis in comparison to a low-fat diet [29, 33]. The high-fat diet was made with a semipurified modified AIN-93 formulation for experimental animals. The diet consisted of 40 percent energy (En%) from carbohydrates, 20 En% from protein, and 45 En% from fats. The major carbohydrate, protein, and fat sources were starch plus sugar, casein (milk protein), and lard (CJ Co., Seoul, Korea), respectively. High-fat diets were supplemented with 1% lyophilized dangguijakyak-san powder, 1% lyophilized dangguijihwang-tang powder, 1% dextrose (control), or 30 *μ*g/kg body weight 17*β*-estradiol + 1% dextrose (positive control) to make the nutrient compositions of the diets the same. They were stored at 4°C. The diet was provided every other day and the amount that was consumed was measured and recorded weekly. The amount of each herbal powder to be consumed (dosage) was calculated by the food intake.

### 2.5. Experimental Design

The fifty OVX rats in 5 groups were randomly allocated to the following groups: (1) 1% lyophilized dangguijakyak-san powder with MIA injection (DJY), (2) 1% lyophilized dangguijihwang-tang powder with MIA injection (DJH), (3) 30 *μ*g/kg body weight 17*β*-estradiol + 1% dextrose with MIA injection (positive control), (4) 1% dextrose with MIA injection (placebo; control), or (5) 1% dextrose without MIA injection (normal control). Each group had 10 animals. All rats had free access to water and to the assigned diet of each group. At the beginning of the study, average body weight of each group was about 280 ± 73 g and the rats in each group had no significant differences in their health.

At 8 weeks of providing the assigned diets, MIA or saline was injected into the right knee of the rats in each group and the assigned diets were provided for an additional 3 weeks. At the end of treatment, the rats were anesthetized with ketamine and xylazine (100 and 10 mg/kg body weight, resp.) and serum was separated from blood collected by the cardiac puncture. Periuterine and retroperitoneum fat pads and uterine were then removed and weighed. After removing right knee, in one-half of the rats in each group articular cartilage from the right knee was isolated and stored at −70°C for biochemical analysis. In the other half of the rats, the right knee was fixed with paraformaldehyde to make a paraffin block.

Serum levels of glucose and insulin were measured with a Glucose Analyzer II (Beckman-Coulter, Palo Alto, CA, USA) and radioimmunoassay kits (Linco Research, Billerica, MA, USA), respectively. Insulin resistance was determined using the homeostasis model assessment estimate of insulin resistance (HOMA-IR) by the following equation: HOMA-IR = fasting insulin (*μ*IU/ml) × fasting glucose (mM)/22.5.

### 2.6. Tail Skin Temperature Measurement and Bone Mineral Density (BMD) Measurement

Tail skin temperature was monitored during the experimental periods during the light period using an infrared thermometer for small rodents (BIO-152-IRB, Bioseb, Chaville, France). Three measurements were made 10 min apart and their average value was used as a single data point.

Body composition including BMD, lean body mass, and fat mass was analyzed with a dual-energy X-ray absorptiometer (DEXA; Norland pDEXA Sabre; Norland Medical Systems Inc., Fort Atkinson, WI, USA). DEXA was calibrated with a phantom supplied by the manufacturer prior to use. The animals were laid in a prone position with posterior legs and hip, knee, and ankle articulations being maintained in 90° flexion with external rotation with tape, after anesthetization with ketamine and xylazine (100 and 10 mg/kg bw, resp.). After scanning leg, hip, and knee areas on the right side, BMD was determined by the DEXA instrument equipped with the appropriate software for BMD assessment in small animals [29]. Fat mass and lean mass were also measured in a similar manner to BMD by DEXA.

### 2.7. Oral Glucose Tolerance

At the 10th week, an oral glucose tolerance test (OGTT) was performed on overnight-fasted animals by orally administering 2 g of glucose/kg body weight. Blood samples were taken by tail bleeding at 0, 10, 20, 30, 40, 50, 60, 70, 80, 90, and 120 min after glucose loading to measure the serum glucose levels with a Glucose Analyzer II (Beckman, Palo Alto, CA). The serum insulin levels were measured at 0, 20, 40, 90, and 120 min using a radioimmunoassay kit (Linco Research, Billerica, MA). The average of the areas under the curves (AUC) for the serum glucose and insulin levels was calculated using the trapezoidal rule.

### 2.8. Progression of Osteoarthritis and Pain-Related Behavior Tests

At 3, 7, 14, and 21 days after MIA injection, the diameters of the knees were measured every week using digital calipers (Mitutoyo, Japan) and gross observation was carefully reported every week by the same trained inspector who was blinded to treatment information throughout the study period. All rats were weighed and assessed for knee joint swelling and gait disturbances in cages where they were allowed to move freely. Swelling and limping were classified as no change (0), mild (1), moderate (2), and severe (3) on the basis of severity [29].

At 7, 14, and 21 days after MIA injection, pain-related behaviors were determined by an incapacitance test using a hind paw limb weight-bearing apparatus (Linton incapacitance tester, UK) and the maximum running speed on a treadmill. Both measurements are a diagnostic criterion for osteoarthritis to demonstrate the intensity of joint discomfort. They can be useful for the discovery of novel pharmacologic agents in human osteoarthritis [34]. The incapacitance equipment is tested for comparing differences in hind paw weight distribution between the right (osteoarthritic) and left (control) limbs. After acclimatizing the test animals for 30 min, the weight distribution onto the hind paw was measured five times for each rat. Using the average of the middle three values percent weight distribution of the right hind paw was calculated with the equation previously reported.

To measure the maximum running speed on a treadmill, rats were acclimated to the treadmill at 40 cm/sec for 1 min and then the speed was increased to 50 cm/sec for 1 min. The speed of the treadmill was subsequently increased by 5 cm/sec every 1 min until the rats could not continue to run and slid to the back of the treadmill. The maximum speed for running was determined when rats were running for 20 sec at the highest speed. All rats were subjected to treadmill test for less than 5 min in each test.

### 2.9. Isolation of Total RNA from Articular Cartilage and Real-Time PCR

Articular cartilage samples from five rats of each group were collected at 28 days after MIA injection. Each cartilage was individually powdered with a cold steel mortar and pestle and then mixed with a monophasic solution of phenol and guanidine isothiocyanate (TRIzol reagent, Life Technologies, Rockville, MD, USA) for total RNA extraction, following the manufacturer's instructions. Total RNA concentration of the extracts was determined using a Lambda 850 spectrophotometer (Perkin Elmer, Waltham, MA, USA) and cDNA was generated from 1 *μ*g total RNA from articular cartilage of individual rats using a superscript III reverse transcriptase kit (Life Science Technology). Five different cDNA were synthesized from each group and each cDNA was used for real-time PCR. Equal amounts of cDNA and primers for specific genes were mixed with SYBR Green mix (Bio-Rad, Richmond, CA) in duplicate and specific genes were amplified using a real-time PCR instrument (Bio-Rad) with optimal thermal cycling conditions described in previous studies. To assess genes associated with inflammation and degradation of articular cartilage, primers were used to detect tumor necrosis factor- (TNF-) *α*, interleukin- (IL-) 1*β*, IL-6, matrix metalloproteinase- (MMP-) 3, and MMP-13 genes as described previously [29]. Cycle of threshold (CT) for each sample was determined. The gene expression levels in unknown samples were quantitated using the comparative CT method (ΔΔCT method). ΔCT was calculated via the following formula: ΔCT = CT (target gene) – CT (endogenous reference gene, *β*-actin). The relative fold changes were calculated by the equation of ΔΔCt = ΔCt_treatment_ − ΔCt_control_. Results were presented as 2^−ΔΔCT^.

### 2.10. Histopathological Analysis of Knee

After the rats were sacrificed, the morphological changes of knee joints were histologically assessed for knee articular bone narrowing, loss of joint region, cartilage erosion, and osteophyte formation [29, 35]. Knee joints were removed, fixed in phosphate-buffered formalin, subsequently decalcified in 10% nitric acid for 72 h, and embedded in paraffin. Five-micrometer sections were made from the paraffin block and stained with hematoxylin and eosin (H-E) and safranin O-fast green. Histopathological changes in each animal were quantitatively expressed by the scoring system of cartilage damage [29]. Cartilage damage was evaluated by the following scoring system: the depth was scored on a scale of 0–5 where 0 was normal; 1 minimal, affecting the superficial zone only; 2 mild invasion into the upper middle zone only; 3 moderate invasion well into the middle zone; 4 marked invasion into the deep zone but not to the tidemark; and 5 severe full-thickness degradation to the tidemark. The extent of tibial plateau involvement and proteoglycan loss was scored as 1 (minimal), 2 (mild), 3 (moderate), and 4 (severe).

### 2.11. Statistical Analysis

Statistical analysis was performed using SAS software version 7 (SAS Institute) and all results are expressed as a mean ± standard deviation (SD). Sample size was calculated using a G power program (power = 0.90 and effect size = 0.50) and sample size of each group was 10. The parameters having multiple measurements at different time points were analyzed with two-way repeated measures ANOVA with time and group as independent variables and interaction term between time and group. The metabolic effects of different groups were performed by one-way analysis of variance (ANOVA) when the results were measured at one time point. Significant differences in the main effects among the groups were identified by Tukey's test at *p* < 0.05.

## 3. Results

### 3.1. Compounds in the Herb Extracts

**Figure 1:**
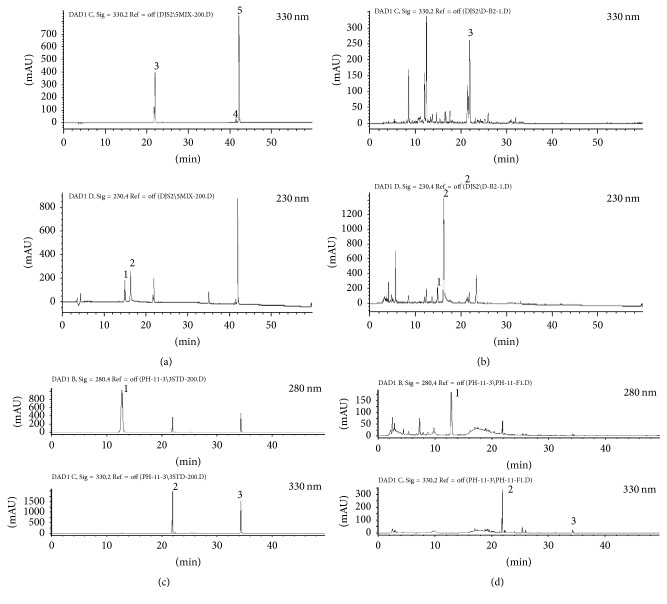
HPLC chromatogram. (a) References for dangguijakyak-san: 1, albiflorin; 2, paeoniflorin; 3, nodakenin; 4, *z*-ligustilide; 5, decursin. (b) Dangguijakyak-san water extract. (c) References for dangguijihwang-tang: 1,5-hydroxymethyl-2-furaldehyde; 2, nodakenin; 3, decursin. (d) Dangguijihwang-tang water extract.

Nodakenin, albiflorin, and paeoniflorin were detected in DJY and paeoniflorin was a major component (Table 1, Figure 1). DJH contained 5-hydroxymethyl-2-furaldehyde, nodakenin, and decursin.

### 3.2. Body Weight and Body Composition

Body weight was higher in the control group than in the positive-control group at the seventh week after the injection of MIA; body weight was lower in the control group than the normal-control group at the eleventh week. The weight loss in the MIA-injected OVX rats was likely to be due to osteoarthritis, which reduced food intake at the seventh week of the experiment (data not shown), but the food intake returned to normal, and gross intake during weeks 9–11 was similar to that in the seventh week (Table 2; *p* < 0.05). Food intake tended to be lower in the positive-control group than the control group at the seventh and eleventh weeks, but it was not significantly different among the groups. Food intake was significantly lower in the positive-control group than the DJY only group at the eleventh week (Table 2). Consumption of the herb extracts was about 148 mg/day for DJY and 144 mg/day for DJH. These amounts of DJY and DJH in rats were equivalent to ~2-3 g per day in humans, when calculated using a conversion coefficient for human doses from experimental animal studies suggested by the US FDA. Periuterine and retroperitoneum fat mass, representing visceral fat, was lower in the control group than the positive-control group and DJY, but not DJH, decreased the visceral fat versus the control group at week 11 of the experimental period (Table 2).

**Figure 2:**
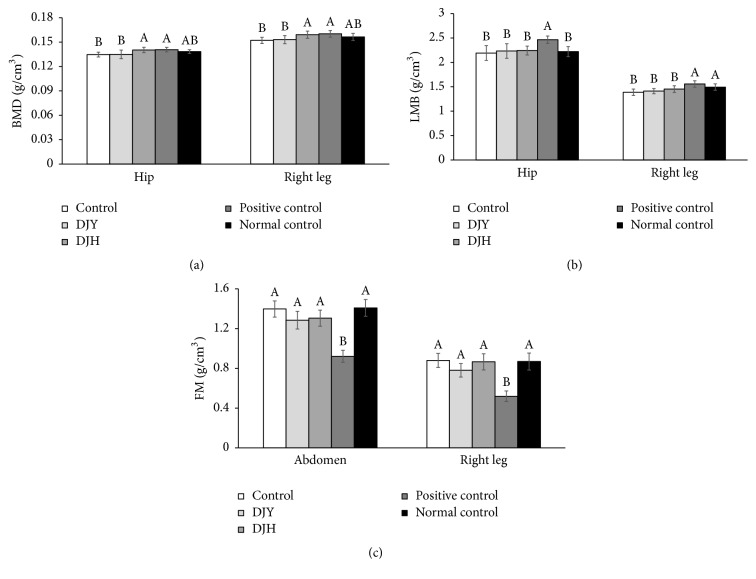
Body composition at the end of experiments. Ovariectomized (OVX) rats were provided with a 45% fat diet containing (1) 1% lyophilized DJY powder + MIA injection (DJY), (2) 1% lyophilized DJH powder + MIA injection (DJH), (3) 30 *μ*g/kg body weight 17*β*-estradiol + 1% dextrose + MIA injection (positive control), (4) 1% dextrose + MIA injection (placebo, control), or (5) 1% dextrose with no MIA injection (normal control). After 4 weeks of the assigned diets, an articular injection of MIA into the right knee was made in all OVX groups except the normal-control group and the assigned diets were provided for an additional 3 weeks. At the 7th week body composition was measured by DEXA. (a) Bone mineral density (BMD). (b) Lean body mass (LBM) at the hip and right leg. (c) Fat mass (FM) at the abdomen and right leg. Each bar and error bar represents the mean ± SD from 10 rats per group. ^A,B^Different letters indicate significant differences in the treatment groups of OVX rats by Tukey's test at *p* < 0.05.

BMD in the hip and right leg was lower in OVX rats than in positive-control rats at the eleventh week of the experimental period (Figure 2(a)). In the normal-control group, with no MIA injection, the BMD of the right leg tended to be higher, but it was not significantly different from the control or positive-control groups, indicating that osteoarthritis further decreased the BMD in the right leg of OVX rats. The DJH, but not DJY, group showed a higher BMD in the hip and right leg than the control group (Figure 2(a)). Lean body mass (LBM) in the hip and the right leg was significantly higher in the positive-control than the control group, whereas LBM of the right leg was lower in the control group than the normal-control group at week 11 (Figure 2(b)). DJH tended to increase LBM in the right leg to a value above that in the control group, but the difference was statistically significant (Figure 2(b)). In contrast to LBM, fat mass was much lower in the positive-control group than the control group but it was not significantly different between the control and positive-control groups (Figure 2(c)). The DJY and DJH groups tended to be lower than the control group but the difference was not significant (Figure 2(c)).

### 3.3. Uterine Weight, Serum 17*β*-Estradiol Levels, and Skin Temperature

Uterine weight was reduced in the control group versus the positive-control group and neither DJY nor DJH altered the uterine weight (Table 2). Serum 17*β*-estradiol levels were lower in the control group than the positive control; neither DJY nor DJH changed the levels. Thus, apparently, neither DJY nor DJH activated estrogen receptors in the uterus (Table 2). Tail skin temperature was significantly higher in the control group than the positive-control group, but neither DJY nor DJH affected the temperature. There were no detrimental effects observed from treatment with either extract during the experimental periods.

### 3.4. Glucose Metabolism

Overnight fasting serum glucose and insulin levels did not differ between control and normal-control groups, indicating that osteoarthritis apparently did not influence glucose metabolism (Table 3). DJY, but not DJH, significantly lowered serum glucose levels versus the control group. Serum insulin levels in the overnight-fasted state were lower in the DJY and DJH groups than in the positive-control group (Table 3). HOMA-IR indicated a state of insulin resistance in overnight fasting; HOMA-IR was much higher in the control group than in the positive-control group. HOMA-IR decreased in descending order: control, normal-control > positive-control, DJY > DJH (Table 3).

**Figure 3:**
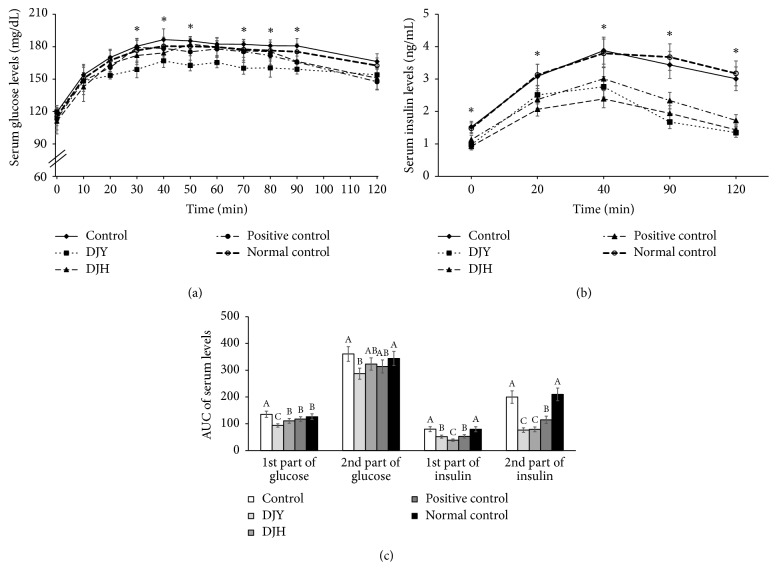
Changes of serum glucose and insulin levels during oral glucose tolerance test at the end of experiment. Ovariectomized (OVX) rats were provided with a 45% fat diet containing (1) 1% lyophilized DJY powder + MIA injection (DJY), (2) 1% lyophilized DJH powder + MIA injection (DJH), (3) 30 *μ*g/kg body weight 17*β*-estradiol + 1% dextrose + MIA injection (positive control), (4) 1% dextrose + MIA injection (placebo, control), or (5) 1% dextrose with no MIA injection (normal control). After 4 weeks of the assigned diets, an articular injection of MIA into the right knee was made in all OVX groups except the normal-control group and the assigned diets were provided for an additional 3 weeks. At 7th week rats were orally given 2 g glucose/kg body weight and serum glucose and insulin levels were measured. (a) Changes of serum glucose levels. (b) Changes of serum insulin levels. (c) Area under the curve of serum glucose and insulin levels. Each bar and error bar represents the mean ± SD from 10 rats per group. ^A,B,C^Different letters indicate significant differences in the treatment groups of OVX rats at each time point identified by Tukey's test at *p* < 0.05.

After giving glucose orally, serum glucose levels reached a peak at 40–50 min and levels at the peak were determined by both insulin secretion and insulin sensitivity (Figure 3(a)). Serum glucose levels decreased from the peak mainly due to insulin sensitivity. Serum glucose levels at the peak were much higher in the control group than the positive-control group and DJY lowered peak levels versus the control group (Figure 3(a)). Serum insulin levels increased until 40 min, and they declined after 40 min (Figure 3(b)). The levels were much higher in the control group than the positive-control group at all time points during the oral glucose tolerance test (OGTT). The DJY group showed lower serum insulin levels than the control group at all time points (Figure 3(b)). The areas under the curves of glucose (AUCGs) and insulin (AUCIs) were separated into two parts (0–40 min and 40–120 min). The reference point was determined by the peak serum insulin levels. The AUCGs of the first and second parts were much greater in the control group than in the positive-control group (Figure 3(c)). The DJY group showed the lowest AUCGs in the first and second parts; DJY also lowered them versus the control but the decrease was less than that with DJY. AUCIs in the first part decreased in descending order, control > DJY = positive-control > DJH, whereas those in the second part were also lower in the positive-control than the control, and both DJY and DJH reduced them (Figure 3(c)). Thus, DJY improved glucose tolerance by improving insulin sensitivity in OVX rats.

During the OGTT, insulin sensitivity at 40 and 80 min was calculated as serum glucose levels divided by serum insulin levels at 40 and 80 min, respectively; higher values indicated better insulin sensitivity. Insulin sensitivity at 40 min was higher in ascending order: control and normal-control < DJY and positive-control < DJH. However, insulin sensitivity at 80 min during the OGTT was lower in the control group than the positive-control group, whereas DJY and DJH increased the values more than the positive control (Table 3). These results suggest that DJY and DJH both improved insulin sensitivity in the fasting and postprandial states more than the positive control.

### 3.5. Global Observations of Osteoarthritis Symptoms

**Figure 4:**
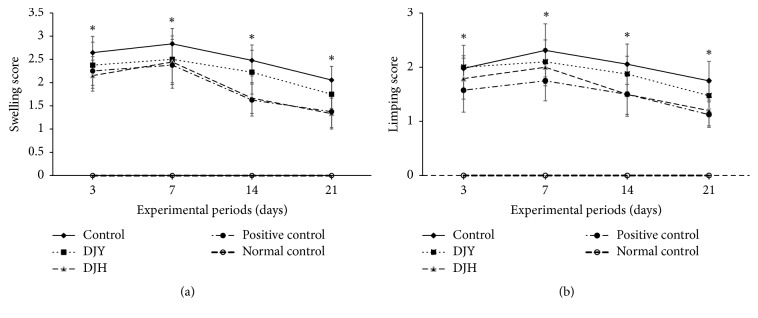
Gross observation of osteoarthritis symptoms at 3, 7, 14, and 21 days after monoiodoacetate (MIA) injection. Ovariectomized (OVX) rats were provided with a 45% fat diet containing (1) 1% lyophilized DJY powder + MIA injection (DJY), (2) 1% lyophilized DJH powder + MIA injection (DJH), (3) 30 *μ*g/kg body weight 17*β*-estradiol + 1% dextrose + MIA injection (positive control), (4) 1% dextrose + MIA injection (placebo, control), or (5) 1% dextrose with no MIA injection (normal control). After 4 weeks of the assigned diets, an articular injection of MIA into the right knee was made in all OVX groups except the normal-control group and the assigned diets were provided for an additional 3 weeks. (a) The scores of the swelling in the right knee. (b) The scores of limping in the right knee. Each data point and error bar represents the mean ± SD from 10 rats per group. ^A,B,C,D^Different letters indicate significant differences in the treatment groups of OVX rats at each time point identified by Tukey's test at *p* < 0.05.

Osteoarthritis developed with inflammation in the joints and showed clinical changes in the right knee, such as limping and swelling (Figure 4). On the day after MIA injection, the right knee began to swell and the rats dragged the leg and limped (Figures 4(a) and 4(b)). The symptoms became more severe until day 7 and then declined generally. With a saline injection, instead of MIA, the normal-control group did not exhibit any swelling or limping. The rats of the positive-control group did not show increased swelling at day 3 in comparison with those of the control group, and the swelling improved in the positive-control group more than in the control group (Figure 4(a)). DJY induced swelling in the right knee from day 3, but not as much as in the positive controls, especially at days 14 and 21. However, the DJH exhibited swelling as badly as the positive-control group. Limping showed similar results to swelling in the right knee (Figure 4(b)).

### 3.6. Pain-Related Behavior Tests for Osteoarthritis

**Figure 5:**
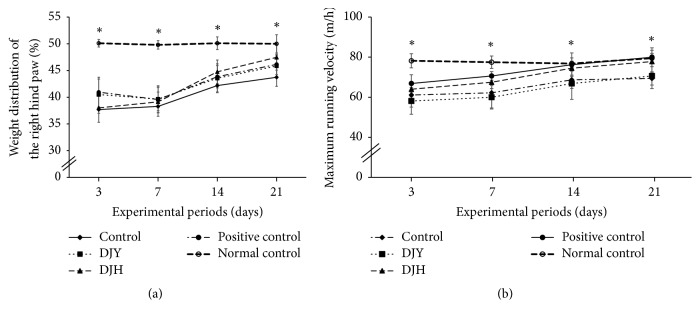
Pain-related behaviors after osteoarthritis induction at 3, 7, 14, and 21 days after monoiodoacetate (MIA) injection. Ovariectomized (OVX) rats were provided with a 45% fat diet containing (1) 1% lyophilized DJY powder + MIA injection (DJY), (2) 1% lyophilized DJH powder + MIA injection (DJH), (3) 30 *μ*g/kg body weight 17*β*-estradiol + 1% dextrose + MIA injection (positive control), (4) 1% dextrose + MIA injection (placebo, control), or (5) 1% dextrose with no MIA injection (normal control). After 4 weeks of the assigned diets, an articular injection of MIA into the right knee was made in all OVX groups except the normal-control group and the assigned diets were provided for an additional 3 weeks. The pain indicators were measured. (a) Differences in weight distribution of the right hind paw (%) measured by an incapacitance tester. (b) Maximum velocity run on a treadmill; each data point and error bar represents the mean ± SD from 10 rats per group. ^A,B,C^Different letters indicate significant differences in the treatment groups of OVX rats at each time point identified by Tukey's test at *p* < 0.05.

Pain-related behavior was measured by the asymmetric weight distribution and maximum velocity of running on a treadmill. Weight distribution on the right hind paw was much lower in the control group than normal-control group, due to pain in the right knee. The weight distribution increased to 50% as the days passed in all rats with MIA injection (Figure 5(a)). The weight distribution was higher in the positive-control group than the control group. DJH increased the weight distribution more than the control group (Figure 5(a)). The maximum velocity in treadmill running decreased as pain increased due to osteoarthritis. The maximum velocity was significantly lower in the rats of the control group versus the normal-control rats (Figure 5(b)). Rats in the positive-control and DJH groups showed greater maximum velocities than those in the control group (Figure 5(b)). Thus, pain-related behaviors in rats with MIA-injected legs were alleviated by DJH by as much as those in the positive-control group.

### 3.7. Levels of Cytokines in the Articular Cartilage of the Right Knee

**Figure 6:**
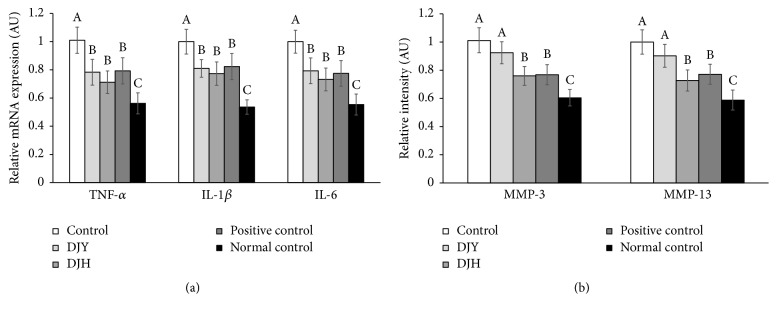
The mRNA expression of proinflammatory cytokines and matrix metalloproteinases (MMPs) in the articular cartilage. Ovariectomized (OVX) rats were provided with a 45% fat diet containing (1) 1% lyophilized DJY powder + MIA injection (DJY), (2) 1% lyophilized DJH powder + MIA injection (DJH), (3) 30 *μ*g/kg body weight 17*β*-estradiol + 1% dextrose + MIA injection (positive control), (4) 1% dextrose + MIA injection (placebo, control), or (5) 1% dextrose with no MIA injection (normal control). After 4 weeks of the assigned diets, an articular injection of MIA into the right knee was made in all OVX groups except the normal-control group and the assigned diets were provided for an additional 3 weeks. The mRNA expression of genes related to collagen degradation and proinflammatory cytokines was measured in the articular cartilage by real-time PCR. (a) mRNA expression of MMP-3 and MMP-13. (b) Proinflammatory cytokines (TNF-*α*, IL-1*β*, and IL-6). Each bar and error bar represents the mean ± SD from 4 rats per group. ^A,B,C^Different letters indicate significant differences in the treatment groups of OVX rats at each time point identified by Tukey's test at *p* < 0.05.

The levels of the proinflammatory cytokines TNF-*α*, IL-1*β*, and IL-6 increased in the articular cartilage of the MIA-injected knees versus the saline-injected knee group. The levels were lower in the positive-control group than the control group. DJH tended to decrease their levels more than DJY but there was no significant difference (Figure 6(a)). Thus, DJH, DJY, and positive-control groups showed lower levels of TNF-*α*, IL-1*β*, and IL-6, but the decrease was not as large as in the normal control.

Levels of MMP-3 and MMP-13 were higher in the articular cartilage of MIA-injected knees in the control group than the positive-control group (Figure 6(b)). DJH significantly inhibited the increase in MMP-3 and MMP-13, down to the levels of the positive-control group (Figure 6(b)). DJY tended to lower the expression of MMP-3 and MMP-13 more than in the positive-control group.

### 3.8. Histopathological Analysis

**Figure 7:**
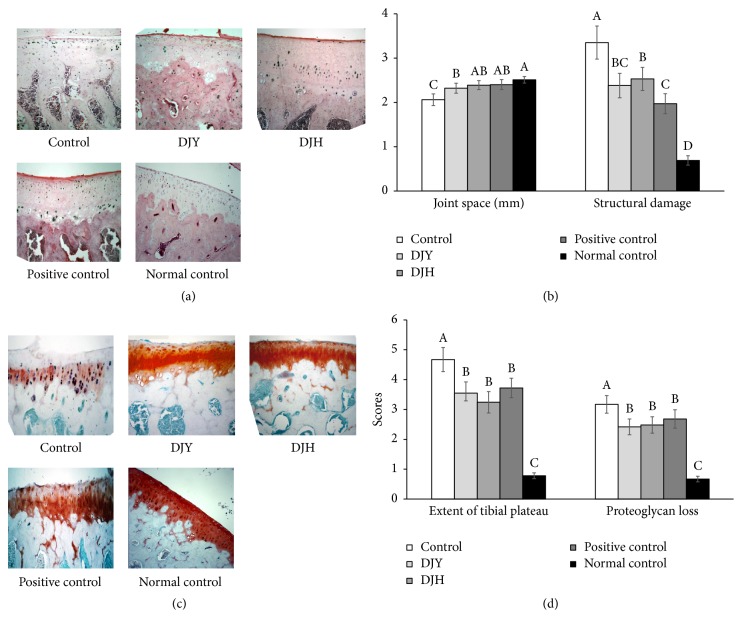
Histopathological features of osteoarthritic lesions in the knee joints of rats at 21 days after intra-articular injection of monoiodoacetate (MIA). Ovariectomized (OVX) rats were provided with a 45% fat diet containing (1) 1% lyophilized DJY powder + MIA injection (DJY), (2) 1% lyophilized DJH powder + MIA injection (DJH), (3) 30 *μ*g/kg body weight 17*β*-estradiol + 1% dextrose + MIA injection (positive control), (4) 1% dextrose + MIA injection (placebo, control), or (5) 1% dextrose with no MIA injection (normal control). After 4 weeks of the assigned diets, an articular injection of MIA into the right knee was made in all OVX groups except the normal-control group and the assigned diets were provided for an additional 3 weeks. (a) Images of H-E staining in the right knee (×10). (b) Quantification of the joint space and structural damage. (c) Safranin O-fast green staining in the right knee (×10). (d) Quantification of cartilage damage. Each bar and error bar represents the mean ± SD from 4 rats per group. ^A,B,C,D,E^Different letters indicate significant differences in the treatment groups of OVX rats at each time point identified by Tukey's test at *p* < 0.05.

Histological evaluations of H-E-stained tissue revealed that MIA injection damaged the articular cartilage and subchondral bone of the knee in OVX control rats (Figure 7). The joint space was narrowed in the control in comparison with the normal control and the space was increased by DJY, DJH, and the positive control to a similar extent. The normal-control rats showed normal structures of the articular cartilage and bone, with smooth articular surfaces, normal chondrocytes, and intact tide marks and subchondral bones. However, MIA injection induced the degeneration of chondrocytes and the tide mark and penetration of subchondral bones. Treatment with 17*β*-estradiol reduced the deterioration in the histological scores of the knees injected with MIA. DJY and DJH also decreased the degeneration of articular cartilage and DJY improved it as much as 17*β*-estradiol treatment in MIA-injected knees (Figure 7(a)).

The tibial plateau was extended in the control in comparison with the normal control and this extension was reduced by DJY, DJH, and the positive control (Figure 7(b)). DJH showed the least extension of tibial plateau but there was no significant difference among DJY, DJH, and 17*β*-estradiol. Proteoglycan loss was much higher in the control group than in the normal-control group. DJY and DJH decreased the proteoglycan loss versus the control group and the decrease was similar to that in the positive-control group (Figure 7(b)). These results indicated that DJY and DJH treatments prevented breakdown of the articular cartilage and the deterioration of bone and joint structures.

## 4. Discussion

DJY and DJH are known to have anti-inflammatory properties and they have been used traditionally to improve health in women and to ameliorate cognitive deficits and depression [36–38]. In this study, the OVX rats showed menopausal symptoms, such as dysregulation of energy, glucose, lipid, and bone metabolism, and increased tail skin temperatures as in previous studies [22, 23]. OVX rats are known to be well representative of human menopausal symptoms [22, 23]. Additionally, osteoarthritis is somewhat exacerbated in an estrogen-deficient state. These menopausal symptoms can be reversed by 17*β*-estradiol treatment. Clinical studies have reported a protection by HRT against diabetes: diabetes risk has been shown to be reduced by 62% in women using HRT in comparison to those having not used HRT. In addition, HRT improves glucose regulation in diabetic women by improving insulin sensitivity and potentiating insulin secretion capacity after menopause [39]. However, the mechanism by which 17*β*-estradiol regulates metabolism is not completely understood [2]. Menopausal symptoms can be reversed by HRT with 17*β*-estradiol. However, HRT has adverse effects such as increasing breast cancer, coronary artery disease, endometrial cancer, venous thromboembolism, and stroke. Thus, there is a continuing need for alternative treatments to attenuate menopausal symptoms.

Many studies have been conducted to assess herbs containing estrogen-like substances. Estrogen acts on several different pathways. Among them, estrogen activates the estrogen receptor- (ER-) *α*, ER-*β*, and G protein-coupled estrogen receptor (GPER) to regulate physiological pathways in various tissues, such as the hypothalamus, liver, bone, and islets [1, 40]. Alternative therapies for alleviating menopausal symptoms need to target the ERs in specific tissues to treat metabolic diseases. In the present study, DJY and DJH were not very effective at improving energy metabolism: DJY reduced only the visceral-fat mass. They also did not prevent the increase in skin tail temperature in OVX rats. However, they did improve insulin sensitivity in fasting and hyperglycemic states. DJY lowered serum glucose levels in the OGTT by increasing serum insulin levels and insulin sensitivity. DJH showed better improvement of insulin sensitivity than DJY in a hyperglycemic state but it did not decrease serum glucose levels during the OGTT by as much as DJY. Thus, DJY can be appropriate for improving glucose metabolism in the menopausal state. This activity may be related to the affinities of DJY and DJH for the ER-*α*, ER-*β*, and GPER that differ in their ligand-binding domains [41]. ER-*α* activation promotes glucose-stimulated insulin biosynthesis, decreases lipid accumulation in the islets, and enhances *β*-cell survival against proapoptotic stimuli, whereas ER-*β* activation potentiates glucose-stimulated insulin secretion [40]. GPER activation also protects *β* cells from apoptosis and promotes glucose-stimulated insulin secretion and lipid homeostasis without affecting insulin biosynthesis. Previous studies have demonstrated that genistein has a much higher affinity for ER-*β* (87% of the affinity of 17*β*-estradiol) than ER-*α* (4% of 17*β*-estradiol) although estradiol has a similar affinity for both estrogen receptors [41]. Different phytoestrogens also have differing affinities for ER-*α* and ER-*β*. DJY and DJH both contain some of the same and some different flavonoids; these flavonoids may have differing affinities for the two ERs [41].

The incidences of hip, knee, and finger osteoarthritis are greater in men than in women before 50 years of age but, after that age, epidemiological studies reveal that they are more common in women than men [42, 43]. This indicates that estrogen deficiency increases the prevalence of musculoskeletal disorders, including osteoporosis and osteoarthritis. This increase is associated with disturbances in joint, cartilage, subchondral bone, synovium, ligaments, muscle, and tendons as a result of estrogen deficiency. However, there have been few interventional studies linking serum levels of other estrogens and estrogen metabolites to the pathogenesis of female osteoarthritis [35, 44, 45]. In the Women's Health Initiative trial, women taking estrogens had significantly less joint replacement, suggesting that estrogen prevents osteoarthritis progression via the pleiotropic activities of estrogen in several tissues [35]. The present study showed that 17*β*-estradiol treatment prevented the decrease in BMD and lean body mass and maintained normoglycemia, which had an indirect effect on delaying the progression of osteoarthritis [46]. DJY and DJH showed some differing effects on BMD, lean body mass, and glucose metabolism. DJY resulted in lower serum glucose levels in the OGTT, with higher serum insulin levels in the hyperglycemic state. However, DJH increased BMD and decreased insulin resistance, even though serum glucose levels were somewhat higher during the OGTT. These results suggest that DJY and DJH might both have beneficial effects on the progression of type 2 diabetes.

Osteoarthritis is characterized by articular cartilage loss, hypertrophic bone changes, osteophyte formation, subchondral bone remodeling, and low-grade synovial inflammation [47]. Clinical symptoms include primarily joint pain, joint tenderness, difficulty in movements, and joint deformities [47]. In the present study, osteoarthritis was induced by the articular injection of MIA injection into the right knee, which resembled osteoarthritis in humans [24]. Previous studies have demonstrated chondrocyte degeneration and necrosis at days 1–7 after MIA injection, increased osteoclasts and osteoblasts in subchondral bone by day 7, focal fragmentation and the collapse of bony trabeculae with fibrosis by day 28, and large areas of bone remodeling by day 56 in histological examinations [32]. Analgesic drugs, such as tramadol, celecoxib, and diclofenac, can reduce pain-related behaviors [32, 48]. Thus, the MIA-injected osteoarthritis model is clinically relevant to humans and is useful for studying therapeutic agents and understanding the pathophysiology. In a previous study, we showed that the clinical symptoms recovered by 3 weeks after MIA injection [29]; thus, the progression of osteoarthritis was determined for 3 weeks in this study. The clinical severity of osteoarthritis symptoms, such as limping, swelling, weight distribution on the right hind paw, and maximum running velocity on a treadmill, was exacerbated until day 7 after MIA injection. The scores for limping and swelling were higher and the weight distribution on the right leg and the maximum velocity on the treadmill were lower in the control group than the positive-control group at days 14 and 21, but the DJH group improved as much as the positive control. However, DJY reduced pain-related behaviors in comparison with the control but not as much as the positive control. These results indicated that DJH has greater efficacy for relieving pain than DJY, although DJY has recently been reported to relieve pain in primary dysmenorrhea [13].

Recent studies have indicated that phytoflavonoids, polyphenols, and bioflavonoids, which are natural compounds in fruits, teas, spices, wine, and vegetables, have the potential to modify osteoarthritis and symptoms based on their anti-inflammatory and anticatabolic actions and protective effects against oxidative stress [49]. Both DJH and DJY have been shown to reduce inflammation and oxidative stress but they have not been studied for treating osteoarthritis. In the present study, DJH and DJY decreased the levels of the proinflammatory cytokines TNF-*α*, IL-1*β*, and IL-6 in articular cartilage versus the control, and the decrease was similar to that in the positive-control group. DJH and DJY improved osteoarthritis symptoms by reducing inflammation in articular cartilage. A histological examination of the right knee showed that the DJH group had less articular cartilage damage near the tidemark and proteoglycan loss than the DJY group, although both prevented damage and proteoglycan loss in comparison with the control. This was related to the expression of MMP-3 and MMP-13, mediators of the breakdown of matrix proteins, in the articular cartilage. The DJY group showed higher MMP-3 and MMP-13 levels than DJH although both DJH and DJY had levels lower than in the control. Specific MMPs, such as MMP-1 (collagenase-1), MMP-3 (stromelysin-1), and MMP-13 (collagenase-3), degrade cartilage proteoglycans and type II collagen, leading to osteoarthritis [50]. The present study indicated that suppression of MMP levels may play an important role in alleviating osteoarthritis in addition to reducing inflammation.

## 5. Conclusions

We showed that DJH protected against OVX-induced osteoarthritis by preventing the decrease in articular cartilage, with reduced levels of MMP-3, MMP-13, and also proinflammatory cytokines in OVX rats with osteoarthritis induced by MIA. Indeed, DJH showed similar antiosteoarthritic activity to 17*β*-estradiol treatment. DJY reduced osteoarthritic symptoms in OVX rats, but the reduction was less than that with DJH. However, DJY improved glucose tolerance in OVX rats. Since the symptoms of the animal model have similar characteristics to menopausal women with osteoarthritis, DJH and DJY may be a potential therapeutic herbal treatment for osteoarthritis and glucose intolerance in postmenopausal women.

## Figures and Tables

**Table 1:** The contents of phenolic compounds and flavonoids (unit: mg/g dry weight).

	Water extract of dangguijakyak-san	Water extracts of dangguijihwang-tang
5-Hydroxymethyl-2-furaldehyde	—	1.20 ± 0.052
Nodakenin	2.20 ± 0.03	1.78 ± 0.041
Decursin	—	0.23 ± 0.003
Albiflorin	3.09 ± 0.16	—
Paeoniflorin	18.5 ± 0.69	—
z-Ligustilide	—	—

Values are means ± SD (*n* = 3).

**Table 2:** Metabolic parameters at the end of experimental periods.

	OVX control (*n* = 10)	DJY (*n* = 10)	DJH (*n* = 10)	Positive control (*n* = 10)	Normal control (*n* = 10)
Body weight at 7th week (g)	399 ± 33^a^	387 ± 28^a^	390 ± 19^a^	367 ± 25^b^	397 ± 30^a^
Body weight at 11th week (g)	376 ± 18^b^	374 ± 42^b^	361 ± 28^b^	346 ± 44^c^	411 ± 35^a^
Food intake at 7th week (g)	15.0 ± 2.0	15.8 ± 1.9	15.2 ± 2.3	14.5 ± 2.7	13.4 ± 1.4
Food intake at 11th week (g)	15.7 ± 2.2^ab^	16.2 ± 1.5^a^	15.7 ± 2.3^ab^	14.1 ± 2.1^b^	16.5 ± 1.9^a^
Periuterine fat (g)	9.9 ± 1.1^a^	8.7 ± 1.2^b^	9.7 ± 1.3^ab^	5.7 ± 0.8^c^	10.5 ± 0.9^a^
Retroperitoneum fat (g)	15.1 ± 2.1^ab^	12.0 ± 1.8^b^	14.0 ± 1.5^a^	7.2 ± 0.9^d^	15.9 ± 1.0^a^
Uterine weight (g)	0.23 ± 0.07^b^	0.22 ± 0.05^b^	0.24 ± 0.09^b^	0.61 ± 0.11^a^	0.24 ± 0.10^b^
Serum 17*β*-estradiol levels (ng/ml)	1.7 ± 0.6^c^	1.8 ± 0.5^c^	1.8 ± 0.6^c^	7.9 ± 1.0^a^	1.8 ± 0.8^b^
Tail skin temperature at 11th week (°C)	29.5 ± 0.8^a^	29.2 ± 0.3^a^	29.1 ± 0.2^a^	27.9 ± 0.2^b^	29.3 ± 0.5^a^

Ovariectomized (OVX) rats were provided with a 43% fat diet containing (1) 1% lyophilized dangguijakyak-san powder with MIA injection (DJY), (2) 1% lyophilized dangguijihwang-tang powder with MIA injection (DJH), (3) 30 *μ*g/kg body weight 17*β*-estradiol + 1% dextrose with MIA injection (positive control), (4) 1% dextrose with MIA injection (placebo; control), or (5) 1% dextrose with saline injection (normal control). Values are mean ± SD. ^a,b,c^Values on the same row with different superscripts were significantly different at *p* < 0.05.

**Table 3:** Glucose metabolism.

	Control (*n* = 10)	DJY (*n* = 10)	DJH (*n* = 10)	Positive control (*n* = 10)	Normal control (*n* = 10)
Serum glucose (mg/dl)	123 ± 9^a^	120 ± 5^a^	110 ± 12^b^	115 ± 8^b^	120 ± 13^a^
Serum insulin (ng/ml)	1.56 ± 0.21^a^	0.97 ± 0.15^c^	0.92 ± 0.13^c^	1.13 ± 0.14^b^	1.48 ± 0.19^a^
HOMA-IR	8.5 ± 1.0^a^	5.2 ± 0.7^b^	4.5 ± 0.6^c^	5.7 ± 0.6^b^	7.9 ± 0.9^a^
Insulin sensitivity at 40 min during OGTT	13.4 ± 1.7^c^	15.8 ± 2.1^b^	19.5 ± 2.4^a^	16.6 ± 2.0^b^	13.0 ± 1.6^c^
Insulin sensitivity at 80 min during OGTT	14.0 ± 1.6^c^	26.5 ± 3.3^a^	26.0 ± 2.9^a^	21.2 ± 2.6^b^	13.0 ± 1.5^c^

Ovariectomized (OVX) rats were provided with a 43% fat diet containing (1) 1% lyophilized dangguijakyak-san powder with MIA injection (DJY), (2) 1% lyophilized dangguijihwang-tang powder with MIA injection (DJH), (3) 30 *μ*g/kg body weight 17*β*-estradiol + 1% dextrose with MIA injection (positive control), (4) 1% dextrose with MIA injection (placebo; control), or (5) 1% dextrose with saline injection (normal control). Values are mean ± SD. ^a,b,c^Values on the same row with different superscripts were significantly different at *p* < 0.05.

## Abbreviations

OVX: Ovariectomy
MIA: Monoiodoacetate
TFA: Trifluoroacetic acid
1% lyophilized DJY: Dangguijakyak-san powder with MIA injection
DJH: 1% lyophilized dangguijihwang-tang powder with MIA injection
Positive control: 30 *μ*g/kg body weight 17*β*-estradiol + 1% dextrose with MIA injection
Control: 1% dextrose with MIA injection
Normal control: 1% dextrose without MIA injection TFA, trifluoroacetic acid
PI3-kinase: Phosphoinositide 3-kinase
MAPK: Mitogen-activated protein kinase
TNF-*α*: Tumor necrosis factor-*α*
HRT: Hormone replacement therapy
SERMs: Selective estrogen receptor modulators
HOMA-IR: Homeostasis model assessment estimate of insulin resistance
BMD: Bone mineral density
DEXA: Dual-energy X-ray absorptiometer
OGTT: Oral glucose tolerance test
MMP: Matrix metalloproteinase
SD: Standard deviation
ANOVA: Analysis of variance
ER-*α*: Estrogen receptor-*α*
GPER: G protein-coupled estrogen receptor.
